# Unilateral Exophthalmos as the First Sign of Chronic Obstructive Hydrocephalus in a Pediatric Patient: A Case Report

**DOI:** 10.7759/cureus.71781

**Published:** 2024-10-18

**Authors:** Mirjana Raicevic, Srdjan S Nikolovski, Sandra Nedovic, Irina Milojevic

**Affiliations:** 1 Department of Neurosurgery, University Children's Hospital, Belgrade, SRB; 2 School of Medicine, University of Belgrade, Belgrade, SRB; 3 Department of Pathology and Laboratory Medicine, Cardiovascular Research Institute, Loyola University Chicago, Maywood IL, USA; 4 Department of Radiology, University Children's Hospital, Belgrade, SRB; 5 Department of Anesthesiology, University Children's Hospital, Belgrade, SRB

**Keywords:** exophthalmos, eyelids, hydrocephalus, orbit, ventriculomegaly

## Abstract

Orbital complications are rare manifestations of congenital hydrocephalus. We present a case of a child presenting with unilateral exophthalmos and palpebral edema as a result of a chronic increased intracranial pressure and severe enlargement of the ventricular system.

The initial presentation in our 13-year-old male patient was progressive right eyelid swelling. Echo-sonography showed right eyelid edema while computed tomography revealed internal hydrocephalus and cerebral edema. In the later course, an enlarging right bulb protrusion occurred, which was repositioned immediately after placing a ventriculoperitoneal shunt. The right eyelid edema decreased in the week following the procedure. The postoperative status was confirmed by magnetic resonance imaging, which also showed a right orbital roof defect.

The observed orbital roof defect was considered a cause of the eye bulb protrusion in this case and highlights the possibility of orbital signs as the first manifestations of non-traumatic obstructive hydrocephalus in children.

## Introduction

The broad definition of hydrocephalus describes it as an increase of the amount of cerebrospinal fluid (CSF) amount within the skull, associated with brain edema [1]. More narrowly, hydrocephalus is a ventricular enlargement that causes accelerated head growth or requires surgical intervention [2]. The most precise one is the definition proposed by the International Hydrocephalus Working Group, which defines hydrocephalus as an active distension of the ventricular system resulting from inadequate passage of CSF from its point of production to the point of absorption [3]. This characterizes hydrocephalus as a progressive disease. Therefore, non-progressive enlargement of the ventricles is excluded from this definition of hydrocephalus.

Globally, the incidence of hydrocephalus diagnosed at birth is approximately 81 per 100,000 live births [4]. The specific incidence of this condition in the territory of Serbia is still unknown due to the lack of epidemiological reports in this area.

Hydrocephalus is most commonly seen in young children and is rare in adults. Symptoms can depend on age, amount of brain damage, and cause of CSF buildup. Symptoms also can vary between infants and older children. In infants, symptoms that can be verified are downward eye gaze, irritability, seizures, sutures diastasis, sleepiness, and vomiting. Older children can manifest changes in personality, changes in facial appearance, loss of eye movement control, feeding difficulties, irritability, sleepiness, nausea and vomiting, headache, urinary incontinence, ataxia, or muscle spasms [5,6].

Hydrocephalus can rarely cause orbital complications. Many studies described prolapse of meninges on the anterior skull wall in children, and most of them were congenital [7]. Some of them described prolapse of the ventricular system but none interfered with orbital space [8].

We present a case of a pediatric patient with unilateral exophthalmos and palpebral edema as a result of chronic increasing intracranial pressure (ICP) and severe enlargement of the ventricular system.

This manuscript was created according to the World Medical Association Declaration of Helsinki. Written informed consent for patient information and images to be published was provided by the patient’s legally authorized representative.

This article was previously presented as a meeting abstract at the 14th Days of BHAAAS (Bosnian-Herzegovinian American Academy of Arts and Sciences) on June 2, 2023.

## Case presentation

A physical exam of a 13-year-old boy presented with progressive swelling of the right eyelids during the three weeks before the exam, which led to the initial diagnosis of cellulitis. Vision tests and motility were normal as well as dilated-pupil fundus examination, ear, nose, and throat examination, and laboratory analyses. Echo-sonography of the right orbit showed physiologic findings except for right eyelid edema. A head computed tomography (CT) scan, besides edema of both right eyelids, also showed mild edema of the right lacrimal gland. Cranial findings revealed internal non-communicating hydrocephalus and cerebral edema.

The patient's family history of neurological and neurosurgical disorders was negative, vital signs and regular blood tests were normal, while genetic testing revealed no chromosomal abnormalities. 

**Figure 1:**
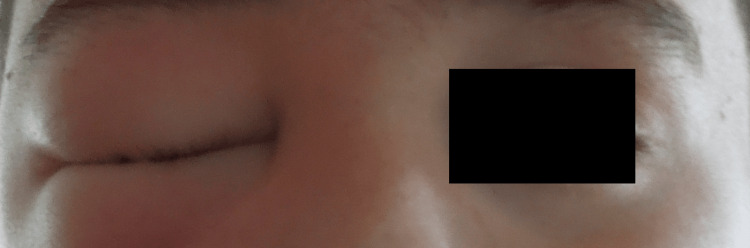
Right eyelid edema seen preoperatively

In the meantime, an enlarging right bulb protrusion out of the right orbital space occurred. Indication for ventriculoperitoneal shunt implantation has been set and the surgery was performed the next day after the CT scan. Preoperative physical exam findings included distinct right eye bulb protrusion out of the orbit with stretched right eyelids, which were displaced forwards (Figure 1). The left eye was normal.

During the surgical intervention, immediately after implanting the cranial catheter, the protruded right eye bulb started repositioning back into the right orbit. There were no complications during the intervention. Previous right eyelid edema was significantly reduced in the following week after surgery.

**Figure 2:**
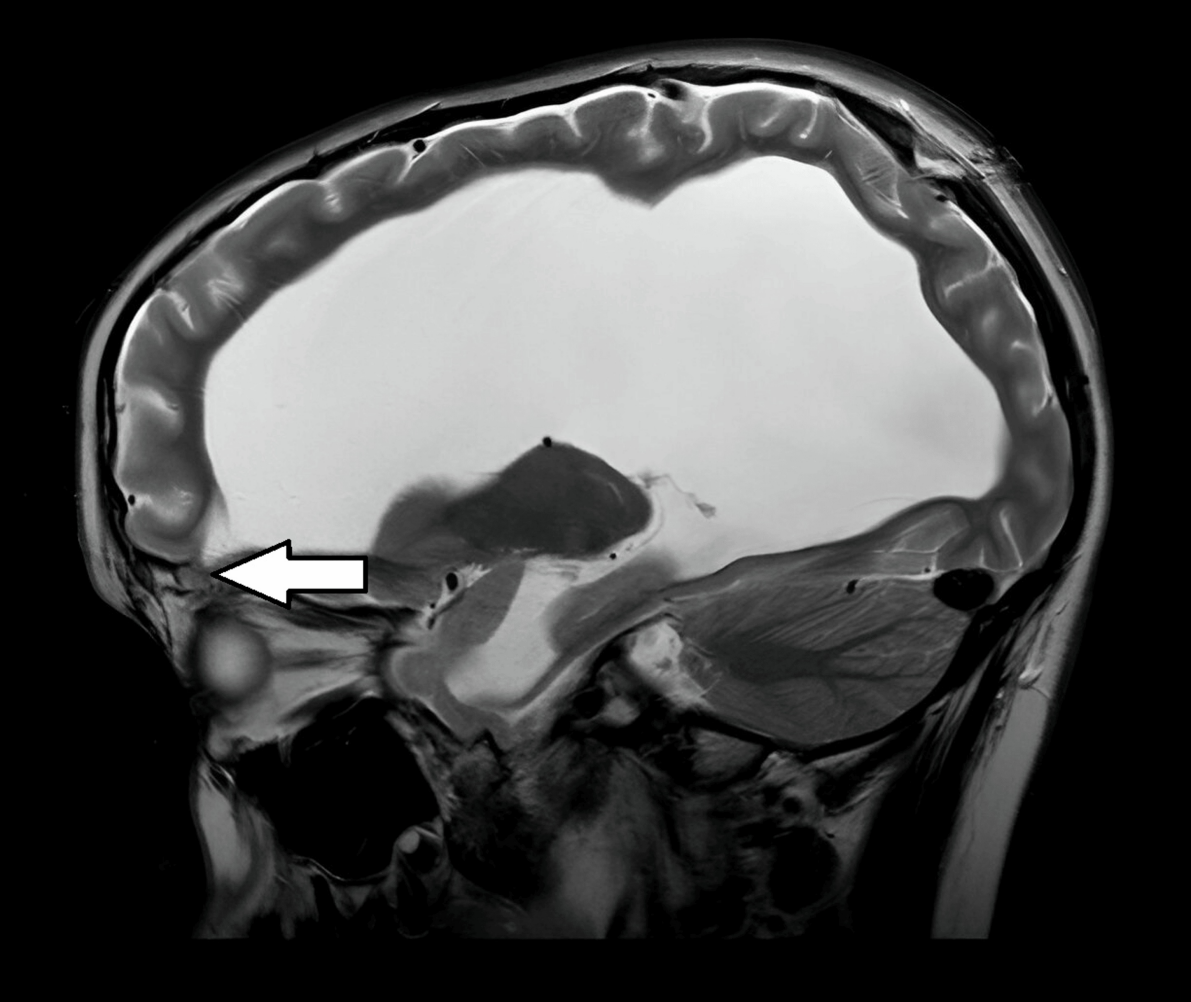
Postoperative magnetic resonance imaging findings (arrow pointing to the herniated meninges through the orbital roof defect)

Postoperative magnetic resonance imaging (MRI) confirmed the reduction of right eyelid edema. It also showed significant dilatation of lateral ventricles with mild wall undulation. The volume of periventricular white matter was significantly reduced, and there were evident signs of diffuse cortical and subcortical atrophy. In some places, there was mild aplasia of cerebral gyri, with minimal subdural collection in the region of the frontal and parietal cortex. Vascular structures showed physiological appearance. However, MRI revealed a defect on the floor of the front cranial fossa, which is equivalent to the right orbit roof, with still present intraorbital herniation of frontal cerebral meninges (Figure 2). The placed ventriculoperitoneal shunt was adequately positioned.

Postoperative control physical exams and MRI revealed regression of all signs and symptoms without any neurological or visual sequelae. One year following the initial procedure, a regular MRI showed dilation of the ventricular system, indicating shunt malfunctioning. At that time, no signs or symptoms associated with the malfunction of the shunt were present, including orbital and vision-related manifestations. A shunt revision was performed leading to ventricular size regression and no further complications (including protrusions through the orbital roof defect) nor shunt malfunction episodes were observed.

During the follow-up after the initial surgery and the shunt revision procedure, no follow-up scores were assessed and the patient received no pharmacological treatment associated with the procedures performed. The patient was followed up for three years after the initial procedure, i.e. two years after the shunt revision.

## Discussion

The presented case shows another rare possible complication of hydrocephalus. Furthermore, this case is even more specific because of eyelid edema and bulb protrusion as possible first notable signs of the presence of hydrocephalus.

From a different point of view, this report draws attention to cases when patients have protrusion of an eye bulb, especially when it is present unilaterally, pointing out that one of the possible causes could be an increase in ICP, although the increase must be extremely high.

Some of the previous reports presented cases of orbital meningo-encephalocele and pulsatile exophthalmos but mostly in posttraumatic conditions and mainly in adults [9-12]. Intra-orbital or periorbital meningo-encephaloceles in pediatric patients have also been described but in most cases as a complication of orbital roof fractures [13,14]. Besides posttraumatic cases, there are also described cases of fetal orbital encephalocele detected during intrauterine development [15] and meningo-encephalocele in pediatric patients due to congenital nontraumatic orbital wall defect [16].

The patient described in this report did not have any pathologic findings on dilated-pupil fundus examination. Besides that, vision tests appeared normal. Therefore, after the surgery, he did not require any examinations considering measurements of optic nerve sheath diameter or orbital volume. The positive postoperative course required only regular control physical examinations and head MRIs.

Considering orbital protrusion of intracranial structures, only a couple of studies presented this situation in patients with hydrocephalus. A report published in 2013 described bulb luxation due to optic nerve sheath enlargement because of chronic hydrocephalus, also in adults [17].

Sharma et al. described a rare case of blepharoencephaloventriculocele in a patient with chronic hydrocephalus [18]. A couple of other studies also described cases of posttraumatic CSF blepharocele in children [19].

Finally, there are only a couple of reports that are very similar to ours regarding the presence of ventricular diverticulum in patients with diagnosed hydrocephalus. One of them is a case report published in 2012, presenting a case of ventricular diverticulum secondary to obstructive hydrocephalus, in a child with occipital encephalocele [8].

The summary of the most important reports of hydrocephalus cases manifesting with orbital signs is presented in Table 1.

**Table 1:** Selected case reports on orbital manifestations of hydrocephalus

Authors	Year	Reference Number	Pediatric/Adult	Pathoanatomic substrate	Orbital manifestation	Posttraumatic
Morihara et al.	2010	[9]	Adult	Orbital meningoencephalocele	Pulsatile exophthalmos	Yes
Hwang et al.	2012	[10]	Adult	Orbital meningoencephalocele	Pulsatile exophthalmos	Yes
Sharma et al.	2014	[11]	Adult	Orbital meningoencephalocele	Pulsatile exophthalmos	Yes
Toktaş et al.	2016	[12]	Adult	Orbital meningoencephalocele	Pulsatile exophthalmos	Yes
Pellant et al.	2010	[13]	Pediatric	Orbital meningoencephalocele	Ptosis, diplopia	Yes
Asil et al.	2015	[14]	Pediatric	Orbital meningoencephalocele	Pulsatile exophthalmos	Yes
Ahmed et al.	2013	[15]	Pediatric	Orbital encephalocele	Complete eye bulb protrusion	No
Hoang et al.	2017	[16]	Pediatric	Orbital meningoencephalocele	Ptosis, blepharedema	No
Shah et al.	2013	[17]	Adult	Optic nerve sheet enlargement	Eye bulb sub-luxation	No
Sharma et al.	2017	[18]	Pediatric	Blepharoencephaloventriculocele	Blepharedema	No
Chandra et al.	2013	[19]	Pediatric	CSF blepharocele	Blepharedema	Yes

In our case, prolapse of the ventricular diverticulum occurred most probably through a perforation of an eroded orbital roof by a hydrocephalic brain. Furthermore, palpebral edema of the right upper eyelid was a consequence of imbibition of the interstitial space with CSF due to increased ICP. Although detailed genetic tests were performed in these patients, chromosomal abnormalities were not detected.

**Figure 3:**
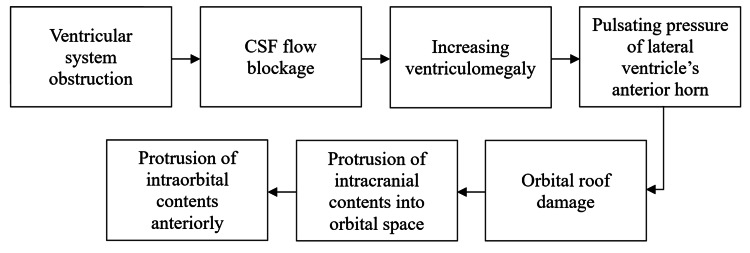
Proposed pathophysiology of orbital manifestations in the presented case CSF: cerebrospinal fluid

The uniqueness of this case lies in orbital signs being one of the primary manifestations of hydrocephalus in this patient, emphasizing the diversity of presentation of this condition. It is clear that the direction of presenting in this case was determined by the localization of ventricular diverticulum protrusion and that it may depend on several different reasons such as the patient's age, previously performed surgical procedures, the presence of another intracranial pathology, etc. There might be weak spots of meninges, which might also be individually distributed, characterized by a higher probability of protrusion in case of increased localized pressure. In this case, the pressure of the enlarging ventricle on the corresponding orbital roof caused further weakening of the meninges and protrusion through the already present bone defect (Figure 3). Therefore, it is very difficult to determine in every patient how increasing ventriculomegaly will manifest, especially because in most cases, such manifestations as orbital protrusion are very rare. On the other hand, presentations like this may shift focus to hydrocephalus since it might be the first presentation in these patients and lead physicians to timely diagnosis and management.

Therefore, this case report highlights the need for neurological assessment for any orbital manifestations, including eye bulb protrusion, whether those are unilateral or bilateral. Additionally, in situations where such rare manifestations of increasing ventriculomegaly occur before the more usual ones as part of the newly developed hydrocephalus, a prompt evaluation and, if needed, reaction is necessary in cases of repetitive similar manifestations during the follow-up period after the initial treatment.

## Conclusions

This report presents a rare case of a pediatric patient with hydrocephalus initially presenting with unilateral eye bulb protrusion, which was previously reported only in adults. It is considered in this case that the orbital roof erosion occurred as a result of chronically increasing intracranial pressure, which consequently caused the unilateral protrusion of the eye bulb, making it the initial sign of hydrocephalus. Its association was confirmed after placing the CSF drainage system and performing the postoperative MRI. This report highlights the possibility of orbital manifestation as the first presentation of chronic obstructive non-traumatic hydrocephalus in children. This report also emphasizes the need for increased attention in these cases to ocular and orbital manifestations in similar cases since those may also be one of the initial signs of ventricular shunt malfunctioning, which could increase the chances of timely management. 
